# Oncofertility in gastrointestinal oncology: a pan-European study of the patient perspective

**DOI:** 10.1093/oncolo/oyag314

**Published:** 2026-08-05

**Authors:** Tomas Sokop, Tal Goshen-Lago, Natali Shirron, Tineke Buffart, Natasha Münch, Radka Obermannova, Veronika Kunesova, Michal Vocka, Dominika Svobodova, Joanna Kufel-Grabowska, Shira Segal-Koperman, Irit Ben-Aharon

**Affiliations:** Department of Comprehensive Cancer Care, Faculty of Medicine, Masaryk University, Brno, 65653, Czech Republic; Division of Oncology, Rambam Health Care Campus, Haifa, 3109601, Israel; Division of Oncology, Rambam Health Care Campus, Haifa, 3109601, Israel; Department of Medical Oncology, Cancer Center Amsterdam, Amsterdam University Medical Center, Amsterdam, 1100 DD, The Netherlands; Digestive Cancers Europe Organization, Brussels, 1040, Belgium; Department of Comprehensive Cancer Care, Faculty of Medicine, Masaryk University, Brno, 65653, Czech Republic; Center of Excellence CREATIC, Faculty of Medicine, Masaryk University, Brno, 62500, Czech Republic; Department of Oncology, First Faculty of Medicine, Charles University and General University Hospital, Prague, 12808, Czech Republic; Department of Oncology, First Faculty of Medicine, Charles University and General University Hospital, Prague, 12808, Czech Republic; Department and Clinic of Oncology and Radiotherapy, Medical University of Gdańsk, Gdańsk, 80-214, Poland; Stop Cancer – Young Cancer Patients Organization, Israel; Division of Oncology, Rambam Health Care Campus, Haifa, 3109601, Israel; Rappaport Faculty of Medicine, Technion, Haifa, 3109601, Israel

**Keywords:** gastrointestinal cancer, fertility preservation, oncofertility, sextual health, young adult cancer patients, cancer survivals

## Abstract

**Introduction:**

The rising incidence of early-onset gastrointestinal (GI) cancer has made the impact of treatment-related gonadal toxicity increasingly significant. While there is ample evidence on oncofertility outcomes in breast cancer and hematological malignancies, data in GI cancers remain limited. We sought to evaluate oncofertility perspectives from the viewpoint of young GI cancer survivors across Europe.

**Methods:**

A cross-sectional 60-item electronic survey regarding oncofertility practices was distributed via patient digital platforms. The target population was survivors treated for GI cancers before the age of 45 or 50 years (women/men). Statistical analysis was performed on country, gender, and tumor type.

**Results:**

One hundred and forty survivors from 19 countries completed the survey (78F/62M), median age at diagnosis was 36.9/35.6 years (F/M), the majority had colorectal cancer, treated with chemotherapy (88%/89%) and radiotherapy (34%/48%). Fertility impact was discussed in 66% of women and 71% of men, but fewer were referred for fertility preservation (43%/57%), and fewer underwent preservation (18%/61%), with variability between countries. The main reason for not preserving fertility was lack of desire of future offspring (women) or lack of referral (men). Sexual health counseling was conducted in less than 50% of patients. Among women, 46% reported persistent amenorrhea and 64% menopausal symptoms. Significant sexual dysfunction was indicated by 69% of women and 83% of men.

**Conclusion:**

Anti-cancer treatment has substantial impact on fertility and sexual function in young GI cancer survivors. Counseling, referral, and uptake vary across countries. Gonadal-related issues remain an unmet need in GI oncology, requiring improved counseling and clinical practice.

Implications for Practice Young survivors of gastrointestinal cancers report a substantial burden of treatment-related reproductive and sexual health complications, while counseling and fertility preservation referrals remain inconsistent across Europe. These findings support integrating fertility and sexual health discussions into routine care, establishing timely referral pathways for fertility preservation, and incorporating multidisciplinary survivorship services to improve long-term quality of life in this growing patient population.

## Introduction

Current epidemiological trends demonstrate a growing incidence of early-onset gastrointestinal (GI) cancers, driven largely by increases in early-onset colorectal cancer (EOCRC), which now accounts for approximately 13% of all colorectal cancer (CRC) cases.^1^^,^^2^ The sharpest increase has been reported among adults aged 20–30 years, which raises unique clinical and survivorship challenges. These younger patients face potential long-term toxicities, complex psychosocial needs, and quality-of-life concerns.^3^ At the same time, improved survival outcomes and the societal shift toward delayed parenthood have intensified attention on the reproductive consequences of cancer therapy. While reproductive outcomes have been extensively studied in women treated for breast cancer and hematologic malignancies, comparable data for GI cancers are scarce. Fertility concerns have been shown to impact medical decisions in a large cohort of young patients with breast cancer followed prospectively.^4^ These studies implicated that a significant portion of the population experiences substantial distress related to infertility, leading some individuals to modify or decline standard treatments such as chemotherapy and endocrine therapy. Data specifically show that fertility issues are a primary driver for non-adherence and the early discontinuation of hormone treatments as women prioritize family planning. These studies indicate that oncologists should provide comprehensive reproductive counseling and better access to preservation strategies throughout the entire care continuum. Nevertheless, the rate of fertility counseling among breast cancer patients has been extensively studied. It depicts a pattern of increased awareness over time and higher referral rates in recent decades compared with earlier years.^4–7^

The American Society of Clinical Oncology (ASCO) 2025 fertility preservation (FP) guidelines provide the most up-to-date, comprehensive framework for counseling and management.^8^ However, much of the evidence supporting FP counseling and patient-reported outcome (PRO) frameworks for sexual function is based upon breast cancer patients. This evidence is often extrapolated to GI cancers, where disease-specific fertility and sexual-health data are comparatively sparse.^8–11^ The treatment for CRC typically involves chemotherapy, surgery, pelvic radiation for locally advanced rectal cancer, and immunotherapy for patients with microsatellite instability-high tumors. The gonadal and sexual effects of these treatment modalities remain to be elucidated in prospective studies and mostly rely on small retrospective cohorts. The impact of oxaliplatin, which constitutes the backbone of chemotherapeutic regimens of CRC, on gonadal function is very limited and indicates an age-dependent treatment-related amenorrhea.^12^^,^^13^

Sexual dysfunction is a key survivorship issue but remains under-reported and inconsistently assessed in quality-of-life studies.^16^ When evaluated directly, especially after rectal cancer surgery, meta-analyses demonstrated high rates of sexual dysfunction, including early postoperative erectile dysfunction. In contrast, retrospective studies on sexual function following radiation consistently highlighted frequent sexual dysfunction morbidity and the need for routine assessment and counseling.^14^^,^^15^

Standard FP includes sperm cryopreservation or testicular sperm extraction in males and embryo/oocyte cryopreservation or ovarian tissue cryopreservation in females.^8^^,^^16^ Adjunctive measures such as ovarian transposition should be considered for patients receiving pelvic radiotherapy. The 2025 ASCO FP guideline supports its use for preserving ovarian function in appropriately selected patients, including those undergoing pelvic radiation for anorectal cancers,^8^ whereas uterine transposition remains experimental. Gonadotropin-releasing hormone agonists (GnRH) have not been studied in the setting of colorectal and other GI cancer treatment protocols, and there is therefore a lack of evidence for their efficacy in this setting.

Reflecting these evidence gaps, our recent pan-European survey of GI oncologists demonstrated substantial variability in FP counseling and referral practices, often misaligned with existing guidance.^11^ Due to the lack of reproductive data, we aimed to explore oncofertility and sexual parameters from the patient perspective, to identify unmet needs and subsequent actionable strategies to improve clinical practice of young patients with GI cancer.

## Methods

### Study design and population

We conducted a cross-sectional, multicenter, international online survey to explore reproductive and sexual health perspectives among young survivors (post-treatment, with no active disease) of GI malignancies. Eligible participants were individuals diagnosed with a GI cancer (ICD-10, C15–C26) between the ages of 18 and 45 or 50 (woman/man) at the time of diagnosis who consented to participate and complete the survey and were treated and recovered before this report to avoid recall bias.

### Questionnaire development and content

A cross-sectional survey was distributed via a global patient representative organization in Europe or through national networks in participating countries to collect data on reproductive and sexual health perspectives from patients. The questionnaire comprised 60 items addressing demographics, basic clinical characteristics, fertility, and sexual health. The survey was conducted electronically between February and July 2025. The questionnaire was additionally reviewed by a patient representative (Hlas onkologických pacientů, Czech Republic) to ensure clarity, relevance, and patient-centeredness of the survey items.

The survey focused on patients’ experiences and conceptions regarding fertility and sexuality following systemic treatment for GI cancer. It was adopted from former studies exploring reproductive and sexual health outcomes in cancer survivors.^17^^,^^18^

Fertility preservation methods explored in the study were in accordance with global guidelines.^10^^,^^19^ Furthermore, we also addressed the use of GnRH analogs (GnRHa) for ovarian suppression in female patients, as well as uterine transposition for female rectal cancer patients anticipated to undergo pelvic radiation, which are considered nonstandard. We queried barriers to initiating FP discussions, including institutional, economic, and patient-related challenges, as well as post-cancer pregnancies and neonatal health. Sexual health discussion and referral to treatment were also assessed. Questions referring to sexual dysfunction requested subjective evaluation graded as follows: no change, mild change, moderate change, and severe change.

Figure S1 depicts the full survey. The survey was delivered electronically and completed online, distributed via social media, patient community platforms, and national networks to maximize reach and sample diversity. Harmonized language versions were produced by professional translators and reviewed by subject-matter experts to ensure conceptual equivalence and consistency across countries.

Due to limited responses from some countries (see Table S1), regional analyses were restricted to countries with more than 10 responses (Czech Republic, Israel, UK, and Netherlands).

## Results

### Patients’ characteristics

A total of 140 GI cancer survivors who received multimodality treatment and are cancer free at the time of the study completed the questionnaire (78 women; mean age at diagnosis, 36.9, and 62 men; mean age at diagnosis, 35.6). Additional baseline characteristics are summarized in Table 1.

**Table 1.** Patient characteristics.

	Female ≤ 45 (*n* = 78)	Male (*n* = 62)
**Mean age (range)**	36.9 (21–45)	35.6 (23–44)
**Diagnosis (*N*, %)**		
** Esophageal cancer**	—	1 (1.6 %)
** Gastric cancer**	10 (12.8 %)	4 (6.5 %)
** Pancreatic cancer**	3 (3.8 %)	4 (6.5 %)
** Bile duct cancer**	—	2 (3.2 %)
** Liver cancer**	—	2 (3.2 %)
** Small intestinal cancer**	2 (2.6 %)	—
** Colon cancer**	41 (52.6 %)	28 (45.2 %)
** Rectal cancer**	21 (26.9 %)	20 (32.3 %)
**Treatment (*N*, %)**		
** Chemotherapy**	69 (88.5 %)	55 (88.7 %)
** Radiotherapy**	27 (34.6 %)	30 (48.4 %)
** Targeted therapy**	7 (9.0 %)	4 (6.5 %)
** Immunotherapy**	10 (12.8 %)	7 (11.3 %)

### Treatment-related reproductive outcomes

#### Discussion regarding treatment impact on fertility

Among patients who have been treated with chemotherapy, 66.2% of women and 71% of men reported receiving counseling on the potential treatment impact on fertility.

#### Fertility preservation

**Figure 1.**
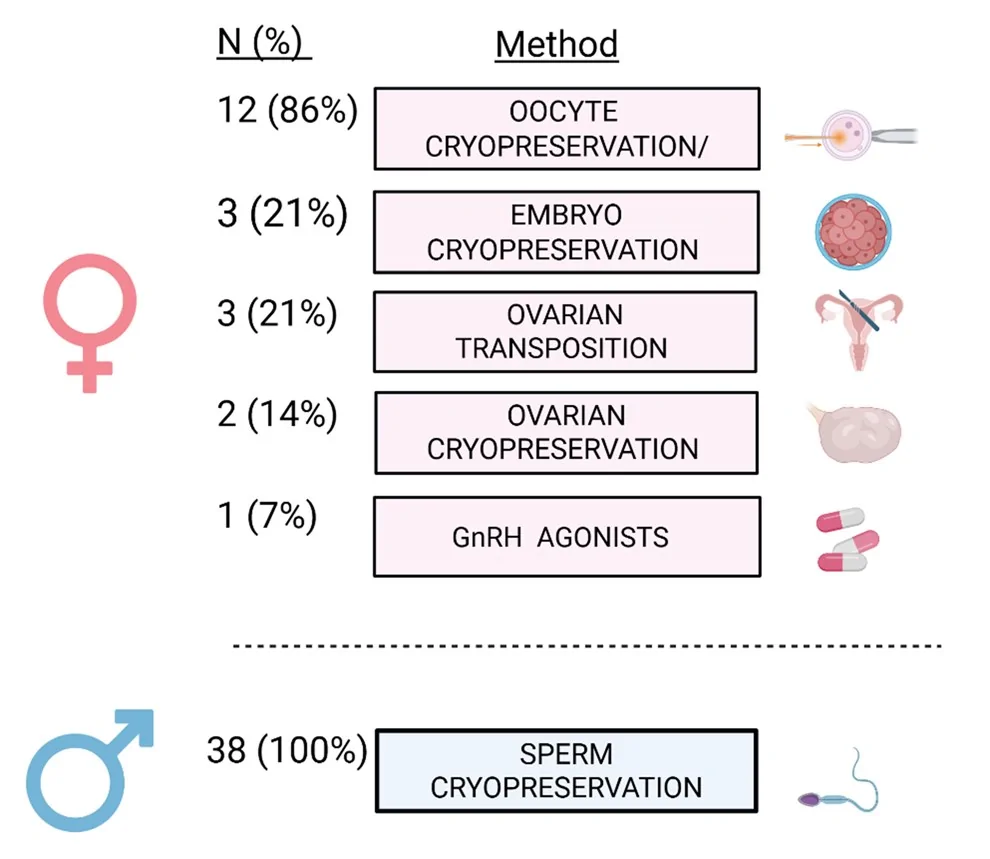
Reported utilization of fertility preservation methods. Numbers represent the report of use for each method. Percentages are calculated out of the total number of participants (women or men) who reported pursuing fertility preservation before treatment initiation.

Referral to FP was reported by 42.9% of women and 57.4% of men. Of these patients, 40% (14/35) of women and 73% of men (38/52) proceeded to FP before treatment initiation. Notably, women younger than 35 years were significantly more likely to undergo FP (10/14, *P* < 0.001). Among the women who reported undergoing FP, 8 underwent oocyte cryopreservation, 3 underwent embryo cryopreservation, 2 underwent ovarian tissue cryopreservation, 2 underwent ovarian transposition, and only 1 used GnRH analogs. All men who reported undergoing FP (*n* = 38) underwent sperm cryopreservation (Figure 1). The main reasons for not proceeding with preservation were a lack of desire for future childbearing (66% in females; 23.8% in males) and concern about treatment delay (14% in females; 28.6% in males); other reasons are summarized in Table S2. There were no significant differences in these reasons by gender or geography.

### Reproductive outcomes reported by responders

#### Menstrual cycle changes

**Figure 2.**
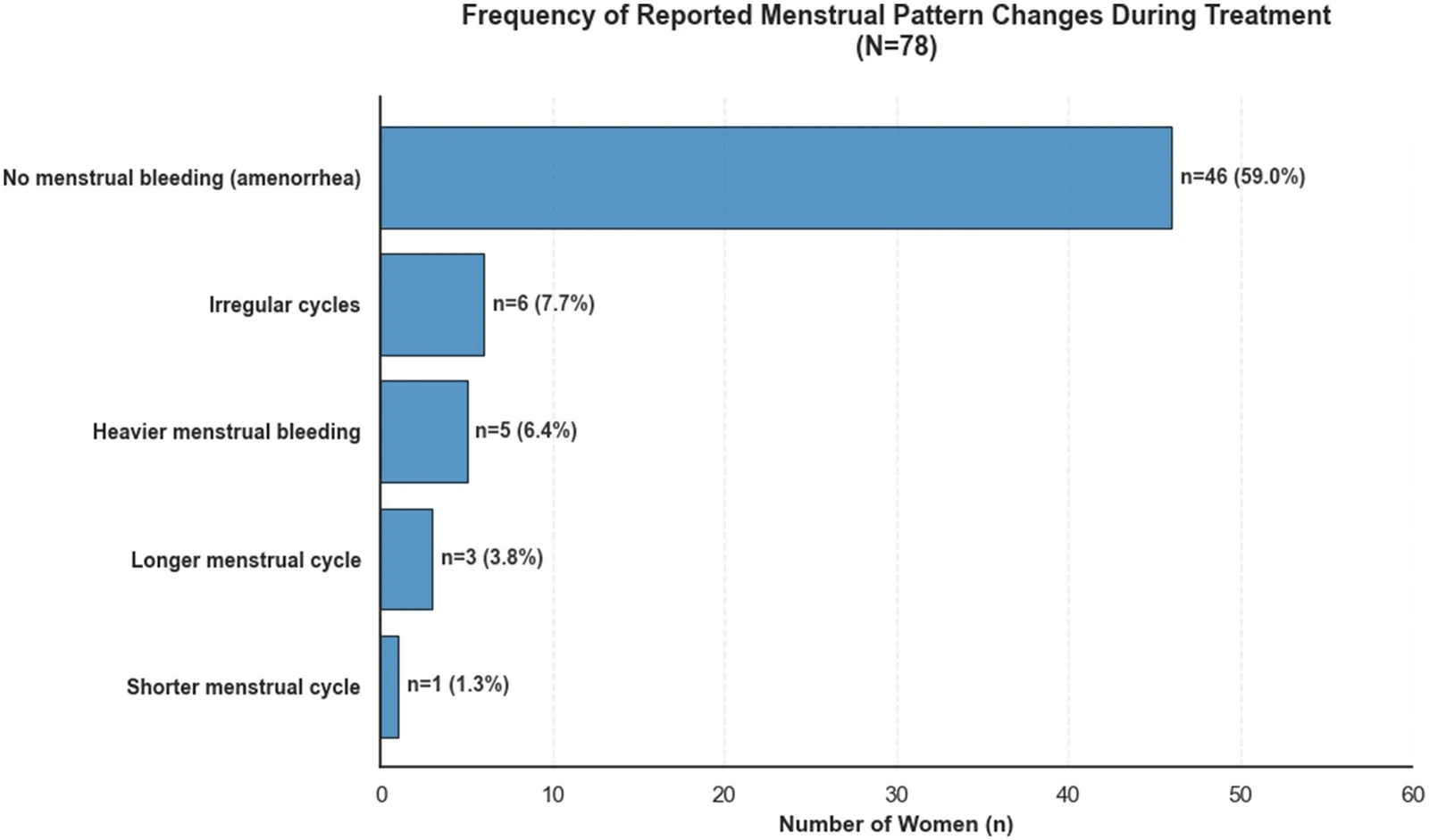
Menstrual pattern alterations during systemic therapy. Distribution of reported menstrual cycle changes among female respondents under age 45 (*n* = 78). Overall, 75.6% (59/78) experienced alterations during treatment, with amenorrhea being the most prevalent (59.0%). Permanent amenorrhea (failure of menses to resume post-treatment) was reported by 44.9% (35/78) of the total cohort. Percentages reflect the proportion of the total female population in this age group.

Among female respondents, 75% (59/78) reported changes in their menstrual cycles during treatment. The most frequent pattern was amenorrhea (46/78, 59%). Forty-four percent of the total population (35/78) indicated that menses did not resume after treatment (ie, permanent amenorrhea). Figure 2 summarizes the frequency of reported changes in menstrual patterns during treatment.

### Impact on sexuality

#### Counseling and outcomes

Discussion and counseling regarding the impact on sexual function was reported by 36.4% of women and 49.2% of men, and counseling on the need for contraception during treatment by 28.6% and 32.2%, respectively. Information was most commonly provided by the clinical oncologist (40.6%).

**Figure 3.**
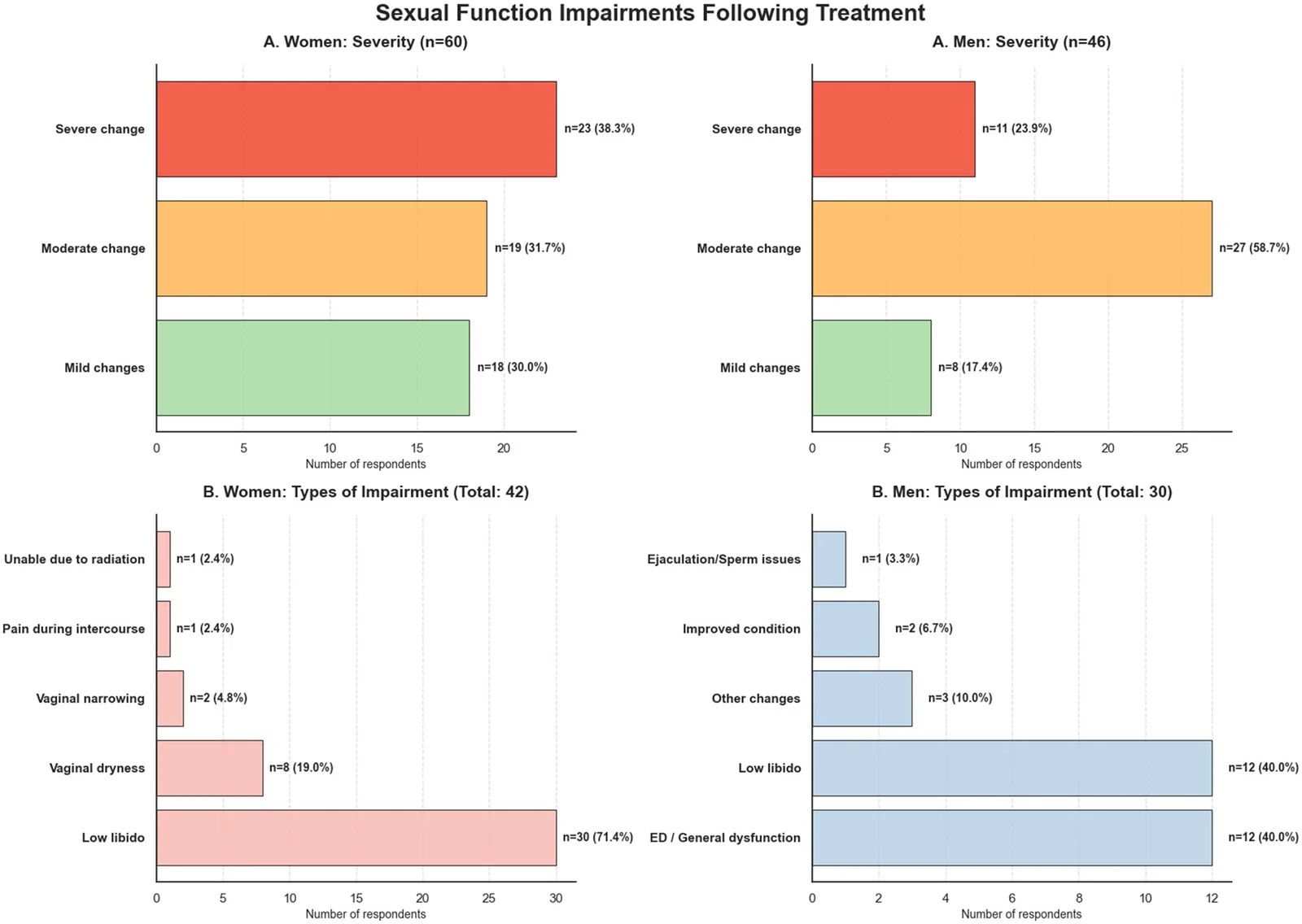
Severity and types of sexual function impairments following treatment. (A) Severity of sexual function impairments: distribution of perceived severity among participants reporting any impairment. Overall, 76.9% of women (60/78) and 74.2% of men (46/62) reported sexual function impairments. Among symptomatic women (*n* = 60), 38.3% reported severe, 31.7% moderate, and 30.0% mild changes. Among symptomatic men (*n* = 46), 23.9% reported severe, 58.7% moderate, and 17.4% mild changes. (B) Types of sexual function impairments: specific concerns reported by participants. The most common issues were low libido in women (71.4%) and erectile/general dysfunction or low libido in men (40.0% each).

Following treatment, most participants reported some degree of sexual function impairment. Among women, 18 (30.0%) reported mild changes, 19 (31.7%) reported moderate changes, and 23 (38.3%) reported severe changes (the total number of women who reported any sexual impairment was 76.9% [60/78]). Among men, 8 (17.4%) reported mild changes, 27 (58.7%) reported moderate changes, and 11 (23.9%) reported severe changes (the total percentage of men who reported any sexual impairment was 74.2% [46/62]). Detailed distributions by severity and impairment types are shown in Figure 3.

### Regional variation in patient-reported counseling and fertility preservation

**Figure 4.**
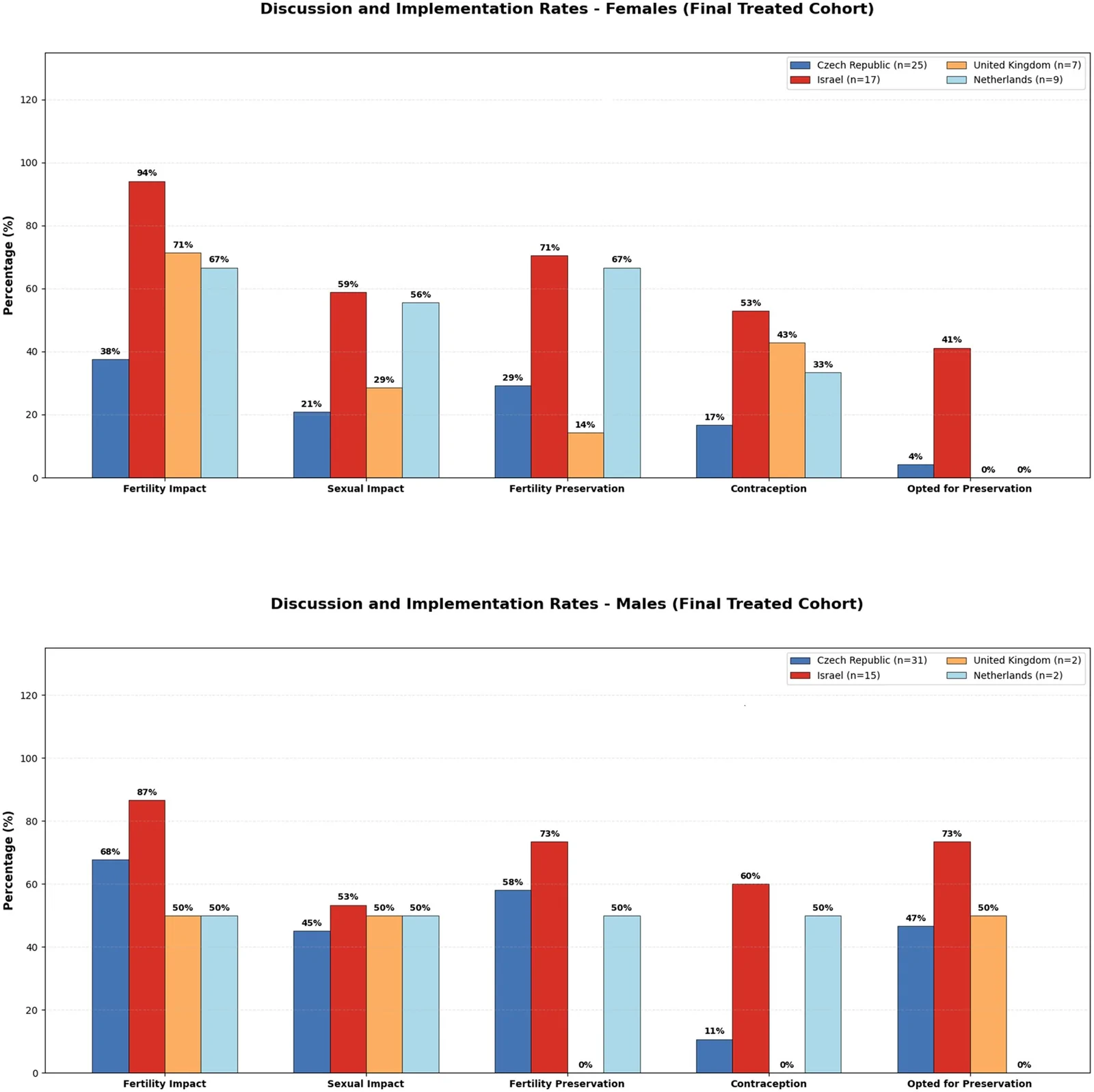
Discussion and implementation of oncofertility and sexual health counseling by country. Percentages represent the proportion of patients within each country who reported receiving information regarding fertility impact, sexual health impact, fertility preservation options, contraception during treatment, or who underwent fertility preservation procedures. Data are presented descriptively to illustrate regional diversity in patient-reported experiences. Results from countries with very small sample sizes, particularly male respondents from the United Kingdom and the Netherlands, should be interpreted cautiously.

Descriptive analyses revealed substantial variation in fertility-related counseling and FP practices across participating countries. Among women, the highest reported rates of fertility impact discussions and FP counseling were observed in Israel, whereas lower rates were reported in other countries. Among men, counseling and FP rates also varied across regions. However, the number of male respondents from the United Kingdom (*n* = 2) and the Netherlands (*n* = 2) was limited; therefore, findings for these countries should be interpreted with caution. Regional data are presented descriptively to illustrate the diversity of patient experiences across participating countries rather than to support formal between-country comparisons. A summary of regional counseling and FP rates is presented in Figure 4.

### Pregnancy outcomes

Four women reported post-treatment pregnancies. Two of these women had also reported treatment-related menstrual changes. Three pregnancies were conceived spontaneously, and one followed in vitro fertilization (IVF), without an obvious signal of adverse neonatal outcomes in this cohort.

Among treated men, 12 reported that their partner gave birth after their treatment cessation, 8 pregnancies were spontaneous, and 3 followed IVF, with 1 response missing regarding conception method. Regarding pregnancy and delivery complications, 1 woman reported a preterm birth at 36 weeks, and there were no reports of low birth weight (<2.5 kg). Two women reported preeclampsia, and 1 reported gestational diabetes. Two male respondents reported developmental disorders among their children, 1 congenital heart defect and 1 case of selective mutism.

## Discussion

Current evidence on reproductive outcomes in the setting of GI cancers is limited to small retrospective cohorts. We aimed to utilize patient/survivor community platforms to reach a larger cohort of young GI cancer survivors to evaluate gonadal and sexual perspectives following systemic curative treatment. Our cohort consisted of 140 young GI cancer survivors who reported that oncofertility issues and sexual dysfunction post-therapy represent a significant unmet need. Notably, menstrual-cycle disruption was reported by 75% of women, with amenorrhea as the most common pattern. Menses did not resume after treatment in about 45% of the cohort, indicating a significant gonadotoxic impact usually accompanied by menopausal symptoms, which may also delineate the high incidence of sexual dysfunction. Thus, compared with former evidence,^12^^,^^13^^,^^19^^,^^20^ the rate of permanent amenorrhea observed in our cohort is significantly higher, suggesting that FP and reproductive counseling are essential in young patients with GI cancers. While resumption of menses and sexual dysfunction are subjective and patient dependent, discussion on FP and sexual health is provider dependent and varies across countries. In our cohort, 66% of women and 71% of men recalled a discussion about potential fertility impairment; 43% of women and 57% of men reported referral to FP, while only 20% of those women and ∼60% of men ultimately underwent FP. The differential figures varied by country and may also be influenced by cultural or societal factors, not only by the availability of the medical system to provide timely assisted reproductive techniques for the rapid process of FP required for cancer patients before commencing systemic treatment. In our previous evaluation of oncologists’ perspectives on FP, we observed a heterogeneous landscape, which is mirrored in this complementary patient-perspective study.^11^

Moreover, fewer than half of the participants recalled counseling about sexual function. In light of the high rate of sexual dysfunction reported by the responders, this represents a clear unmet need with suboptimal care.^8^^,^^16^^,^^21^

Our results denote that when sexual health counseling did occur, information was most commonly provided by the treating oncologist and less by a dedicated professional sexual health specialist or a multidisciplinary service (eg, sexual medicine, pelvic-floor therapy, gynecology/urology, and psycho-oncology). The high incidence of sexual dysfunction is in accordance with former studies depicting a similar trend.^14^^,^^15^^,^^22^^,^^23^ Marked heterogeneity by country/region suggests that structural and cultural drivers may shape care delivery. We observed substantial regional variability in counseling, referral, and uptake, especially among women (differences were significant across all items assessed) and among men (differences in key domains: fertility impact discussion, FP referral, and undertaking FP). Several factors may contribute: (i) differences in awareness and prioritization,^24^^,^^25^ (ii) evidence gaps and extrapolation from other tumor types, and (iii) differences in access, reimbursement, and streamlined referral pathways across health systems.^11^ Within our surveyed countries, public/statutory coverage for medically indicated FP (at least sperm and/or oocyte cryopreservation, with eligibility and storage rules varying) as of to date is documented in Belgium,^26^ the Czech Republic,^27^ France,^28^ Germany,^28^ Israel,^29^ the Netherlands,^30^ Spain,^31^ Sweden,^28^ Poland,^32^ Portugal,^28^ and the UK.^28^ Patient preferences also matter: “no desire for future childbearing” was the most frequently reported reason among women. At the same time, men more often cited that FP was not discussed and concerns about treatment delay, suggesting that communication and timing may be particularly modifiable barriers in male survivors.

Despite a high rate of permanent amenorrhea, post-treatment pregnancies were reported by 4 women. Three pregnancies were conceived spontaneously, and 1 followed IVF, without an obvious signal of adverse neonatal outcomes in this cohort. Current best-practice guidance supports the feasibility of pregnancy after cancer with individualized timing and multidisciplinary oversight, while prospective GI-specific data remain limited.^21^ These observations reinforce the value of early counseling for patients who are interested or uncertain at diagnosis, consistent with guideline recommendations to refer them for FP.^8^

There are several implications for further practice and research derived from our study, as successfully implemented in the setting of breast cancer, oncofertility perspectives should be emphasized also in the setting of young curative GI cancer patients. Moreover, sexual health should be integrated into the clinical practice of supportive programs for young patients with GI cancers. Ideally, these services should be delivered through a multidisciplinary approach rather than relying solely on the treating oncologist. Importantly, discussions around sexual health and FP should begin at the time of diagnosis. Initiating these conversations early ensures that patients of all genders have adequate time to process information and make informed decisions regarding preservation options. Due to the paucity of data and in view of the increased incidence of CRC in young population, it is prudent to establish a prospective study of reproductive and sexual outcomes in this unique population which may shed light on treatment-induced gonadotoxicity, and hopefully also on the role of measures to reduce toxicity as GnRH analogs in this setting, since treatment protocols are different from those of breast cancer, upon which GnRHa were studied. Dedicated programs for multidisciplinary care for young cancer patients may serve as an optimal venue to study these outcomes and interventions.^33^

Strengths of this study include multinational participation, a focus on GI cancer, and PROs that explicitly captured sexual function and oncofertility domains, which are often omitted from clinician-only surveys. However, several limitations warrant consideration. The survey is cross-sectional and subject to recall bias. In addition, the survey did not capture the exact interval between treatment completion and questionnaire completion, which may influence interpretation of menstrual recovery outcomes. Sexual dysfunction severity was self-reported (mild/moderate/severe) rather than measured with validated instruments, reducing comparability across studies. Selection bias is plausible because recruitment via digital patient communities may over-represent engaged survivors, and specialized centers may over-estimate counseling rates relative to routine practice.

In conclusion, with early-onset GI cancers rising, implementation barriers rather than lack of guideline clarity may represent the dominant challenge. Young GI cancer survivors report a high burden of gonadal-related issues and sexual impairment, alongside uneven counseling and FP access across regions. Aligning everyday GI oncology workflows with fertility-preservation and survivorship guidelines while strengthening multidisciplinary sexual-health and menopause-focused supportive care represents a concrete opportunity to improve survivorship care for this growing patient population.

## Supplementary Material

oyag314_Supplementary_Data

## Data Availability

Data generated by the study will be made available upon request.
