# Supplementary material for: Oncofertility in gastrointestinal oncology: a pan-European study of the patient perspective
Source: Oncologist. 2026 Aug 5;31(9):oyag314. doi: 10.1093/oncolo/oyag314 (PMC13503032; doi:10.1093/oncolo/oyag314)
Supplement: Supplementary file 1 [file oyag314_supplementary_data.zip › Supplementary Table S1.docx]

Supplementary Table S1. Distribution of Survey Responses by Country

| **Country** | **Responders Count** |
| --- | --- |
| Czech Republic | 72 |
| Israel | 35 |
| United Kingdom | 14 |
| Netherlands | 14 |
| Poland | 8 |
| Germany | 7 |
| Romania | 5 |
| Belgium | 4 |
| Spain | 2 |
| Sweden | 2 |
| Slovenia | 2 |
| Portugal | 2 |
| United States | 1 |
| Latvia | 1 |
| Azerbaijan | 1 |
| Aruba | 1 |
| Slovakia | 1 |
| Albania | 1 |
| France | 1 |

*The single respondent from the United States was treated in Europe and completed the survey through a participating patient network.
