# Supplementary material for: Oncofertility in gastrointestinal oncology: a pan-European study of the patient perspective
Source: Oncologist. 2026 Aug 5;31(9):oyag314. doi: 10.1093/oncolo/oyag314 (PMC13503032; doi:10.1093/oncolo/oyag314)
Supplement: Supplementary file 1 [file oyag314_supplementary_data.zip › Supplementary Table S2.docx]

Supplementary Table S2. Reasons for Not Pursuing Fertility Preservation

| **Group** | **Reason** | **Count** | **Percent** |
| --- | --- | --- | --- |
| Female ≤45 | No desire for future childbearing | 33 | 66.0% |
|  | Fear of treatment delay | 7 | 14.0% |
|  | Other | 6 | 12.0% |
|  | Not relevant due to age | 2 | 4.0% |
|  | Medical concerns | 1 | 2.0% |
|  | Cost/Financial concerns | 1 | 2.0% |
| Male | Not discussed with the healthcare provider | 6 | 28.6% |
|  | Fear of treatment delay | 6 | 28.6% |
|  | No desire for future childbearing | 5 | 23.8% |
|  | Cost/Financial concerns | 2 | 9.5% |
|  | Other | 2 | 9.5% |
