# Supplementary material for: Oncofertility in gastrointestinal oncology: a pan-European study of the patient perspective
Source: Oncologist. 2026 Aug 5;31(9):oyag314. doi: 10.1093/oncolo/oyag314 (PMC13503032; doi:10.1093/oncolo/oyag314)
Supplement: Supplementary file 1 [file oyag314_supplementary_data.zip › Supp. Figure1_Fertility survey.pdf]

In order to submit this form, you should open it with Adobe Acrobat Reader.

Fertility outcomes in Gastrointestinal Cancer Survivors

please provide your country of residence *

[illegible]

Country

Gender *

- ◯ Male
- ◯ Female

Diagnosis and treatment

Which diagnosis were you treated for? *

- ◯ Oesophageal Cancer
- ◯ Gastric Cancer
- ◯ Liver Cancer
- ◯ Biliary Tract Cancer
- ◯ Small Intestine Cancer
- ◯ Colon Cancer
- ◯ Rectal Cancer
- ◯ Pancreatic Cancer

What was your age when you were diagnosed? *

Years

Please select all treatment modalities you have undergone

- ☐ Surgery
- ☐ Chemotherapy
- ☐ Radiation therapy
- ☐ Targeted therapy
- ☐ Immunotherapy
- ☐ I do not know

Have you been treated or followed up for any non-oncologic diagnosis before the oncological diagnosis (i.e., background morbidity as diabetes mellitus, allergy, psoriasis)?

- ◯ Yes
- ◯ No

If answered "yes," please specify:

Do you suffer from any chronic post-oncological treatment adverse effects (long-term side effects following your cancer treatment)?

◯

Ye
s

○ No

If answered "Yes" Please specify:

[ ]

Quality of fertility and sexual functions consultation

Was the impact of treatments on fertility discussed with you?

○ Yes, before the treatment
○ Yes, during the treatment
○ Yes, before and during the treatment
○ No

Was the impact of treatment on sexual functions discussed with you?

○ Yes, before the treatment
○ Yes, during the treatment
○ Yes, before and during the treatment
○ No

Have you been consulted about fertility preservation before the treatment initiation?

○ Yes
○ No

Have you been consulted about fertility preservation methods options?

○ Yes
○ No

Who provided you with this information?

○ Medical Oncologist
○ Radiation Oncologist
○ Surgeon
○ Fertility Specialist
○ No one
○ Other [ ]

Have you been discussed the importance of contraception methods during the treatment?

○ Yes
○ No

Fertility Preservation

Have you opted for any of the fertility preservation methods?

- ◯ Yes
- ◯ No

Fertility preservation method for men:

- ◯ Sperm cryopreservation
- ◯ Other ____________

Fertility preservation method for female (Choose multiple if necessary):

- ☐ Oocyte cryopreservation
- ☐ Ovarian tissue cryopreservation
- ☐ Embryo cryopreservation
- ☐ Ovarian transposition
- ☐ GnRH analogs (ovarian suppression treatment)

If not, what was the reason?(Choose multiple if necessary)

- ☐ Fear from treatment delay
- ☐ Fear from ovarian stimulation
- ☐ Fear from congenital defects after oncology treatment
- ☐ Fear from pregnancy complications after oncology treatment
- ☐ Other ____________

If not, what was the reason?

- ☐ Fear from treatment delay
- ☐ Other ____________

Menstrual cycle changes

Have your menstrual cycles changed since the first symptoms of the disease occurred?

- ◯ Yes
- ◯ No

Have your menstrual cycles changed since the treatment initiation?

◯ Yes
◯ No

Have your menstrual cycles resumed since the treatment?

◯ Yes
◯ No

Have you experienced premature menopause symptoms? (hot flushes, mood changes, night sweats, vaginal dryness)

◯ Yes
◯ No

If you experienced premature menopause symptoms, please specify:

Sexual Function

Did you experience any changes in your sexual function?

◯ No Change
◯ Mild Change
◯ Moderate change
◯ Severe Change

If possible, please specify which changes you experienced.

Pre-Disease Pregnancies History

Number of previous pregnancies before cancer diagnosis

Previous abortions

◯ Yes
◯ No

Post-treatment Pregnancies

Did you give birth following oncology treatment?

- ○ Yes
- ○ No

Was the pregnancy achieved using in vitro fertilization?

- ○ Yes
- ○ No

Did you experience any pregnancy or delivery complications?

- ○ Yes
- ○ No

If you experienced any pregnancy or delivery complications, please specify:

- ☐ Premature birth
- ☐ Miscarriage
- ☐ Bleeding after delivery
- ☐ Preeclampsia
- ☐ Emergency cesarean section
- ☐ Bleeding prior to delivery
- ☐ Assisted vaginal delivery
- ☐ Other ________

If premature birth (<37 gestational weeks), please specify in what week of pregnancy:

____

Week

If low birth weight (<2.5 kg), please specify:

____

Kg

Did your baby suffer from any postnatal complications? (e.g. need for resuscitation or serious infection)

- ○ Yes
- ○ No

If answered "Yes" Please specify:

____

Does your baby have any development disorder or chronic illness?

- ○ Yes

○

N
o

If answered "Yes" Please specify:

Submit
